# Transient receptor potential ankyrin 1 in spinal cord dorsal horn is involved in neuropathic pain in nerve root constriction rats

**DOI:** 10.1186/1744-8069-10-58

**Published:** 2014-09-06

**Authors:** Tsuyoshi Miyakawa, Yoshinori Terashima, Tsuneo Takebayashi, Katsumasa Tanimoto, Takehito Iwase, Izaya Ogon, Takeshi Kobayashi, Noritsugu Tohse, Toshihiko Yamashita

**Affiliations:** Department of Orthopaedic Surgery, Sapporo Medical University School of Medicine, S1 W16, Sapporo, Hokkaido, 060-8543 Japan; Department of Cellular Physiology and Signal Transduction, Sapporo Medical University School of Medicine, S1W16 Sapporo, Hokkaido Japan

**Keywords:** Transient receptor potential ankyrin 1 (TRPA1), Neuropathic pain, Root constriction, Spinal cord, Substantia gelatinosa neuron, *In vivo* patch-clamp

## Abstract

**Background:**

Lumbar radicular pain is categorized as a type of neuropathic pain, but its pathophysiological mechanisms are not fully understood. The substantia gelatinosa (SG) in the spinal cord dorsal horn receives primary afferent inputs and is considered to be a therapeutic target for treating neuropathic pain. *In vivo* patch-clamp recording is a useful procedure for analyzing the functional properties of synaptic transmission in SG neurons. Transient receptor potential ankyrin 1 (TRPA1) has been widely identified in the central and peripheral nervous systems, such as in the peripheral nociceptor, dorsal root ganglion, and spinal cord dorsal horn and is involved in synaptic transmission of pain. However, its functional role and mechanism of pain transmission in the spinal cord dorsal horn are not well understood. The purpose of this study was to use *in vivo* patch-clamp analysis to examine changes in the excitatory synaptic transmission of SG neurons treated with TRPA1 antagonist and to clarify the potential role of TRPA1 in the rat spinal cord dorsal horn.

**Results:**

The rats with root constriction (RC) showed mechanical hypersensitivity, hyperalgesia, and thermal hyperalgesia. In addition, pin pricks elicited pain-related behavior even in the sham and naïve rats. These pain-related behaviors were significantly attenuated by intrathecal injection of a TRPA1 antagonist. The degrees of intrathecal injection efficacy were equivalent among the 3 groups (RC, sham, and naïve groups). In an electrophysiological study, the frequencies and amplitudes of excitatory postsynaptic currents (EPSCs) were significantly increased in the RC rats compared with those in the sham and naïve rats. Spontaneous EPSCs and evoked-EPSCs by non-noxious and noxious stimuli were significantly decreased by TRPA1 antagonist. As in the behavioral study, there were no statistically significant differences among the 3 groups.

**Conclusion:**

These data showed that the TRPA1 antagonist had an inhibitory effect on mechanical hypersensitivity and hyperalgesia as well as on physiological pain transmission in the spinal cord dorsal horn. This suggests that TRPA1 is consistently involved in excitatory synaptic transmission even in the physiological state and has a role in coordinating pain transmission.

## Background

Lumbar radicular pain is one of the most common symptoms caused by lumbar disc herniation or lumbar spinal canal stenosis. Radicular pain has characteristic symptoms, such as spontaneous pain, allodynia, and hyperalgesia, which are felt in the gluteal region, thigh, leg, and foot. In addition, radicular pain is difficult to relieve and develops into chronic neuropathic pain. Recently, there have been many investigations of neuropathic pain [1]. The substantia gelatinosa (SG) in the spinal cord dorsal horn receives primary afferent inputs, which predominantly convey nociceptive sensations. Nociceptive information is integrated and modified in SG. Therefore, SG may be a therapeutic target for treating neuropathic pain.

*In vivo* patch-clamp recording [2, 3] is an electrophysiological procedure used to observe the tiny membrane currents and voltages of SG neurons. It is a useful procedure for analysis of the functional properties of synaptic transmission in response to naturally applied non-noxious and noxious stimuli because diverse synaptic connectivity is preserved. We previously used *in vivo* patch-clamp analysis and showed that nerve root injury proximal to the dorsal root ganglion (DRG) led to characteristic excitatory synaptic transmission in SG neurons and changed sensory processing in SG neurons [4]. The changes in synaptic transmission led to spontaneous pain, mechanical allodynia, and hyperalgesia contributing to the pathogenesis of radicular pain.

Because transient receptor potential vanilloid 1 (TRPV1), which is a capsaicin receptor, was reported to be involved in pain transmission [5], an interest in temperature-sensitive transient receptor potential (TRP) channels has increased significantly [6, 7]. Transient receptor potential ankyrin 1 (TRPA1) is a calcium-permeable non-selective cation channel [8, 9]. TRPA1 functions as a polymodal nociceptor and can be activated in vitro by mechanical, osmotic, thermal, and chemical stimuli [9–13]. TRPA1 has been widely identified in the central and peripheral nervous systems, such as in the peripheral nociceptor, DRG, and spinal cord dorsal horn [9, 14–16]. Numerous studies have shown that TRPA1 is involved in mechanical hyperalgesia, allodynia, and pain hypersensitivity in the peripheral and central mechanisms [17–20]. TRPA1 was reported to involve excitatory synaptic transmission of glutamic acid from the central terminal of primary afferent fibers. Expression of TRPA1 is upregulated in the spinal cord dorsal horn and nociceptors by spinal nerve injury [18, 21]. Therefore, it is likely that inhibition of TRPA1 in the spinal cord dorsal horn decreases the excitability of SG neurons and that pain transmission or hyperalgesia in the neuropathic pain is consequently attenuated. Some reports have stated that TRPA1 agonists act at both the pre- and postsynaptic terminals [16, 19, 20]. On the other hand, others have stated that TRPA1 antagonists act only at pre-synaptic terminals in the spinal cord dorsal horn [22]. The functional role of TRPA1 regarding the mechanism of pain transmission is not well understood in the spinal cord dorsal horn.

The purpose of this study was to examine changes in the excitatory synaptic transmission of SG neurons treated with a TRPA1 antagonist and to determine the role of TRPA1 in physiological or neuropathic pain transmission in the spinal cord dorsal horn. We performed a behavioral study that used a pin-prick test [23], which provokes pain in the physiological state (non-hypersensitivity state), and performed an electrophysiological study that used *in vivo* patch-clamp recording [2, 3], which can observe the electrophysiological changes in SG neurons caused by physiological stimuli and drugs.

## Results

### Radicular pain

**Figure 1:**
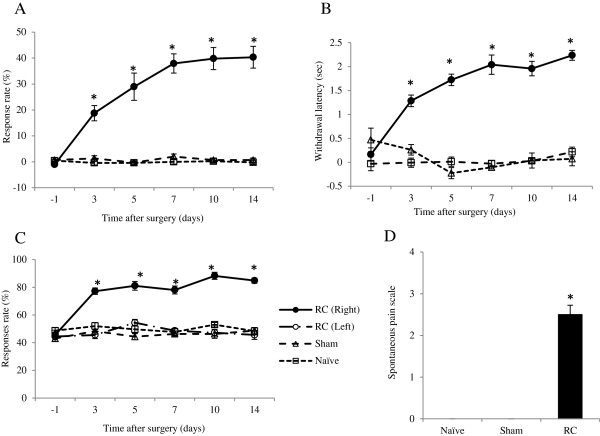
**Time course for mechanical hypersensitivity, thermal hyperalgesia, mechanical hyperalgesia, and spontaneous pain. (A)** A 3.2-g von Frey filament was used to evaluate mechanical hypersensitivity. Root constriction (RC) rats (black circles) showed mechanical hypersensitivity from day 3–14 after the nerve root injury compared with sham rats (white triangles) and naïve rats (white squares). **(B)** The differential score was used to evaluate thermal hyperalgesia. The differential score was significantly higher in the RC rats than in the sham rats and naïve rats from day 3 thereafter. **(C)** The pin-prick test was used to evaluate mechanical hyperalgesia. RC rats showed significantly higher withdrawal responses than the others from day 3 thereafter. In addition, the injured side in the RC rats (black circles), sham rats, and naïve rats and the noninjured side in the RC rats (white circles) also showed withdrawal responses. Error bars denote the standard error of the mean. *P < 0.05 compared with sham and naïve rats; one-way factorial measures of analysis of variance followed by the Tukey–Kramer test. **(D)** The numerical scale (see text) at day 14 was used to evaluate the degree of spontaneous pain-related behavior. All RC rats showed abnormal positioning of the lesioned hind paw, whereas all sham rats and naïve rats showed normal positioning of the hind paw. *P < 0.05 (Mann–Whitney *U* test).

In the behavioral study, the rats with root constriction (RC) showed mechanical hypersensitivity, mechanical hyperalgesia, and thermal hyperalgesia. Figure 1A shows the time course of the response rate to non-noxious mechanical stimuli using a 3.2-g von Frey filament in the RC group (n = 15), the sham group (n = 15), and the naïve group (n = 15). The response rate in the RC group were significantly increased from 3 days after the nerve root injury and thereafter. Those in the sham group and naïve group were not increased. Similarly, the differential score for noxious thermal stimuli was significantly higher in the RC group than in the sham and naïve groups (Figure 1B). In the physiological state (sham and naïve groups), pin prick elicited pain-related behavior. The response rate for noxious pain stimuli using the pin-prick test was significantly higher in the RC group than in the sham and naïve groups (Figure 1C).

Spontaneous pain evaluated on a numerical scale was significantly higher in the RC group than in the sham group and naïve group at 14 days after nerve root injury (Figure 1D). In the sham group and naïve group, all rats showed normal positioning of the hind paw (n = 15, scale = 0, each group). In the RC group, all rats exhibited abnormal positioning of the lesioned hind paws, and the median scale number was 2.5.

### Effects of a TRPA1 antagonist on pain-related behavior

**Figure 2:**
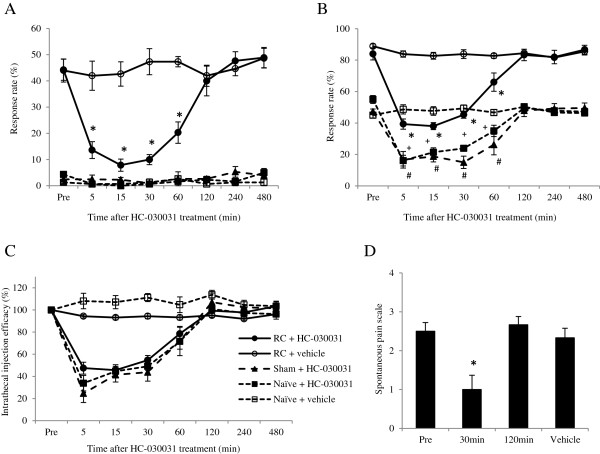
**Effects of HC-030031 on root constriction (RC)-induced mechanical hypersensitivity, hyperalgesia, and spontaneous pain.** The time course for mechanical hypersensitivity and mechanical hyperalgesia in the RC rats, sham rats, and naïve rats after HC-030031 treatment (10 μg, intrathecally). **(A)** The response rate evaluated by a 3.2-g von Frey test was significantly decreased by HC-030031 treatment in the RC rats but did not change in the sham rats and naïve rats. Intrathecal injection of vehicle had no effect in any of the groups. **(B)** The response rate evaluated by pin-prick test was significantly decreased by HC-030031 treatment in the RC rats, sham rats, and naïve rats. Intrathecal injection of vehicle also had no effect in any of the groups. *P < 0.05 compared with RC + vehicle, ^+/#^P < 0.05 compared with naïve + vehicle; one-way factorial measures of analysis of variance followed by Tukey–Kramer test. **(C)** Intrathecal injection efficacy was calculated by dividing the response rate after HC-030031 treatment by the response rate at baseline. There was no statistical difference among the RC rats, sham rats, and naïve rats. **(D)** The degree of spontaneous pain-related behavior was significantly decreased after HC-030031 treatment. Pre-From = before drug injection. *P < 0.05 (Mann–Whitney *U* test).

Intrathecal injection of HC-030031, a TRPA1 antagonist, at a dose of 10 μg significantly decreased the response rate to the von Frey test and pin-prick test and decreased the spontaneous pain scale number. The response rate elicited by the von Frey filament was significantly decreased when mechanical hypersensitivity was assessed between 5 and 60 minutes after HC-030031 treatment in the RC group (Figure 2A). On the other hand, no changes were observed in the sham and naïve groups. The response rates elicited by pin prick were also significantly decreased when the RC group, sham group, and naïve group were assessed between 5 and 60 minutes after HC-030031 treatment (Figure 2B). There were no significant differences in intrathecal injection efficacy against responses to the pin-prick test among the 3 groups (Figure 2C). The spontaneous pain scale number in the RC group was also significantly decreased at 30 minutes after HC-030031 treatment (Figure 2D).

### *In vivo*patch-clamp recording at baseline

A total of 175 SG neurons were recorded at the L5 segmental level of the spinal cord: 63 SG neurons in the RC group, 47 SG neurons in the sham group, and 65 SG neurons in the naïve group. No significant differences in the mean depths of the recording sites were observed from the surface of the spinal cord, resting membrane potential, or input resistance of the SG neurons among the 3 groups (data not shown). The recordings were usually obtained from SG neurons in a stable condition for 10–30 minutes, and stable recordings were maintained in several neurons for more than 5 hours.

### Excitatory postsynaptic currents in SG neurons after nerve root injury

**Figure 3:**
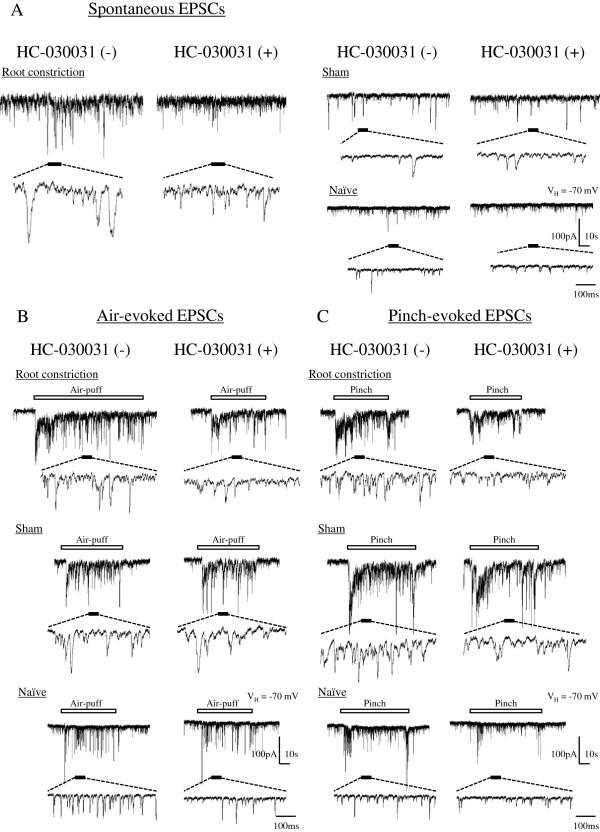
**HC-030031 decreases the frequencies and amplitudes of spontaneous EPSCs.** Representative traces from *in vivo* patch-clamp recording show the spontaneous EPSCs in the absence (left) or presence (right) of HC-030031 (100 μM) of spontaneous EPSCs **(A)**, air-evoked EPSCs **(B)**, and pinch-evoked EPSCs **(C)**. The frequencies and amplitudes of spontaneous EPSCs were significantly higher in the root constriction (RC) rats than in the sham rats and naïve rats. The frequencies and amplitudes were significantly decreased after HC-030031 treatment in all groups of rats.

All SG neurons exhibited spontaneous excitatory postsynaptic currents (EPSCs), and the SG neurons that had a receptive field produced large-amplitude (>50 pA) barrages of EPSCs in response to air-puff or pinch stimuli to the skin of the gluteal region or hind paw at a holding potential (V_H_) of -70 mV. The mean frequencies and amplitudes of spontaneous EPSCs in the SG neurons were higher and larger in the RC group (10.6 ± 0.7 Hz and 23.2 ± 0.8 pA, respectively; n = 63) than in the sham group (8.0 ± 0.8 Hz and 20.5 ± 0.7 pA, respectively; n = 47) and naïve group (8.0 ± 0.6 Hz and 19.2 ± 0.6 pA, respectively; n = 65) (P < 0.05) (Figure 3).

### Effects of a TRPA1 antagonist on EPSCs

**Figure 4:**
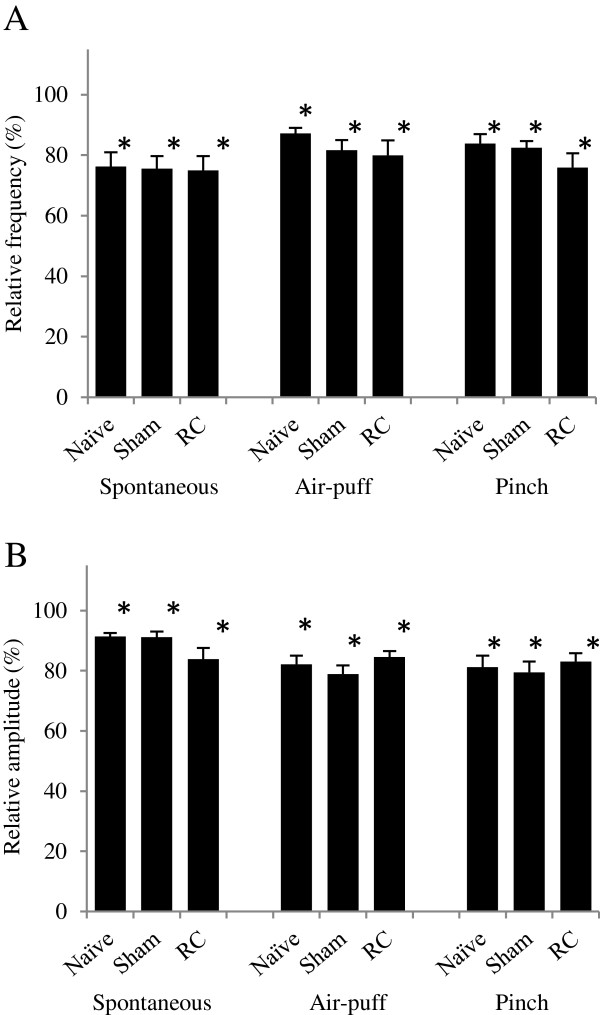
**Changes of EPSCs in SG neurons after HC-030031 treatment.** The relative frequencies **(A)** and amplitudes **(B)** of spontaneous EPSCs and evoked-EPSCs by air-puff or pinch stimuli were significantly decreased after HC-030031 treatment (100 μM) in the root constriction (RC), sham, and naïve groups. There were no significant differences in the relative frequencies and amplitudes among the 3 groups. *P < 0.05 compared with the frequencies and amplitudes before HC-030031 treatment in the respective groups (Student’s paired *t* test).

HC-030031 decreased the frequencies and amplitudes in 10 of 11 recorded SG neurons in the RC group, 11 of 12 in the sham group, and 10 of 11 in the naïve group. In all 3 groups, the frequencies and amplitudes of both the spontaneous EPSCs and evoked EPSCs were significantly decreased after HC-030031 treatment. In the root constriction group, the frequency and amplitude of spontaneous EPSCs were decreased (75.0 ± 4.8% of the control frequency and 83.9 ± 3.6% of the control amplitude) within 30 seconds and those of evoked EPSC were also decreased (79.9 ± 5.0% of the control frequency and 84.6 ± 1.9% of the control amplitude by air-puff stimuli, 75.9 ± 4.8% of the control frequency and 83.1 ± 2.8% of the control amplitude by pinch stimuli). In the sham group, the frequency and amplitude of spontaneous EPSCs were decreased (75.6 ± 4.2% of the control frequency and 91.1 ± 1.9% of the control amplitude) and those of evoked EPSCs were also decreased (81.6 ± 3.4% of the control frequency and 78.9 ± 2.9% of the control amplitude by air-puff stimuli, 82.4 ± 2.2% of the control frequency and 79.5 ± 3.6% of the control amplitude by pinch stimuli). And in the naïve group, the frequency and amplitude of spontaneous EPSCs were decreased (76.2 ± 4.8% of the control frequency and 91.4 ± 1.2% of the control amplitude) and those of evoked EPSCs were also decreased (87.2 ± 1.8% of the control frequency and 82.2 ± 2.9% of the control amplitude by air-puff stimuli, 83.8 ± 3.1% of the control frequency and 81.2 ± 3.8% of the control amplitude by pinch stimuli). There were no statistically significant differences in the relative frequencies and relative amplitudes of the spontaneous EPSCs, EPSCs evoked by air-puff stimuli, and EPSCs evoked by pinch stimuli among the 3 groups (Figure 4).

## Discussion

In the present study, to assess the functional role of TRPA1 in the spinal cord dorsal horn, we evaluated behavioral changes after intrathecal injection of a selective TRPA1 antagonist and electrophysiological changes assessed by *in vivo* patch-clamp recording. The following main results were found: (1) A TRPA1 antagonist attenuated pain-related behavior elicited by noxious pain stimuli in the physiological state. (2) A TRPA1 antagonist attenuated mechanical hypersensitivity and hyperalgesia caused by non-noxious and noxious stimuli and attenuated spontaneous pain-related behavior in the neuropathic pain state. (3) A TRPA1 antagonist reduced the frequencies and amplitudes of spontaneous EPSCs in approximately 90% of SG neurons in the spinal cord dorsal horn.

### The role of TRPA1 in excitatory synaptic transmission elicited by non-noxious and noxious stimuli

Some studies have shown that mechanical hypersensitivity against the von Frey filament was manifested in inflammatory and chemical pain due to injection of Complete Freund’s Adjuvant, formalin, and mustard oil to the hind paw. The mechanical hypersensitivity was inhibited by intrathecal or intraplantar injection of a TRPA1 antagonist [24–27]. In addition, other studies have shown that mechanical hypersensitivity in the neuropathic pain model of chronic constriction injury or spinal nerve ligation (SNL) was inhibited by intrathecal injection of a TRPA1 antagonist [27]. There have been few behavioral studies on the involvement of TRPA1 in noxious stimuli in the physiological state. In an electrophysiological study, McGaraughty et al. reported that a TRPA1 antagonist decreased firing to pinch stimuli but did not decrease firing to tactile stimuli in spinal wide dynamic range neurons [28]. Some reports have used thermal stimuli for evaluating noxious stimuli in non-neuropathic pain models [24, 26, 27, 29]. Because TRPA1 is activated by cold stimuli <17°C, the evaluation of TRPA1 regarding pain transmission may not have been accurate. We used a pin-prick test [23], in which pain-related behavior could be observed in not only the neuropathic pain state but also the physiological state to evaluate pain-related behavior after exposure to noxious pain stimuli.

A TRPA1 antagonist decreased the frequency of pain-related behavior elicited by non-noxious and noxious stimuli in the RC group, sham group, and naïve group, but there were no significant differences among the 3 groups. Similarly, the frequencies and amplitudes of EPSCs evoked by pinch stimuli obtained from *in vivo* patch-clamp recording were decreased by a TRPA1 antagonist in all 3 groups, and the degree of decrease among the 3 groups did not significantly differ. Therefore, it is suggested that TRPA1 is involved in physiological pain transmission in the spinal cord dorsal horn and is not particularly involved in neuropathic pain. In addition, the decreases in the frequencies and amplitudes of the spontaneous EPSCs caused by the TRPA1 antagonist indicate that TRPA1 is always active in the spinal cord dorsal horn at a constant rate without any stimuli.

It is well known that the voltage-dependent calcium channel in the central end of primary afferent fibers is activated when action potential from peripheral nociceptors is reached. Both TRPA1 and the voltage-dependent calcium channel cause calcium to flow into a cell and release the neurotransmitter glutamic acid from synaptic vesicles to the synaptic cleft. Thus, TRPA1 may consistently cause excitability of SG neurons and pain transmission in the spinal cord dorsal horn.

### The role of TRPA1 in the spinal cord dorsal horn in neuropathic pain

Intrathecal injection of a TRPA1 antagonist significantly affected pain-related behavior in the RC group and normal pain-related behavior in the sham group and naïve group. However, there were no significant differences in the behavioral study and electrophysiological study results among the 3 groups. Thus, TRPA1 appeared to be active in the neuropathic pain state and physiological state. Obata et al. reported that expression of TRPA1 was downregulated in the L5 segment and upregulated in the L4 segment when only the L5 nerve root was cut distal to the DRG in SNL model rats [18]. In addition, Kim et al. reported that dorsal rhizotomy caused downregulation of TRPA1 expression at the same segment in the spinal cord dorsal horn [16]. When inflammatory or nerve injury occurred distal to DRG, nerve growth factor increased in inflamed or injured tissue and was transported to DRG; subsequently, TRPA1 was produced in the DRG and transported to peripheral nociceptors and the central end of primary afferent fibers [18, 30, 31]. Thus, when the spinal cord and DRG were unconnected because of a dorsal rhizotomy, the expression of TRPA1 decreased in the central end of primary afferent fibers [16, 18]. We used radicular pain models in which nerve roots were injured proximal to DRG. No previous study has evaluated the expression of TRPA1 in the spinal cord dorsal horn in the same pain model. In previous slice patch-clamp recording studies, 30%–60% of recorded cells were affected by a TRPA1 agonist [19, 20]. On the other hand, in the present study, we observed synaptic transmission of SG neurons by *in vivo* patch-clamp recording, and approximately 90% of the recorded cells were affected by a TRPA1 antagonist. Because the membrane current of SG neurons was recorded under *in vivo* conditions, abundant synapses were completely preserved, and the TRPA1 antagonist affected many cells, in contrast to the methods used in the previous studies.

Degeneration occurs in primary afferents of the L5 nerve root after L5 nerve root injury, which results in a decrease in the number of synapses between primary afferents and spinal cord dorsal horn neurons [32]. This could explain why the effect of the TRPA1 antagonist was not increased after nerve root constriction proximal to DRG. After nerve root injury, TRPA1 production increased in DRG, whereas the number of synapses was decreased because of degeneration. Therefore, the degree of TRPA1 expression may not have changed in the spinal cord dorsal horn. There is another possibility that inactivated TRPA1 increase after nerve root constriction. Immunostaining of TRPA1 expression may be required in further study.

### Possibility of TRPA1 as a therapeutic target for pain

Bautista et al. reported that bradykinin activated TRPA1 by phospholipase C activation and produced peripheral sensitization in which thermal hyperalgesia developed. The authors expected to observed clinical effects from use of TRPA1 antagonists [29].

TRPA1 is activated by mechanical, thermal, and chemical stimuli [10–12, 29] and expressed in the auditory organs, respiratory tract, and gastrointestinal tract [33–36]. When TRPA1 is inhibited, the side effects in each of these organs become a major problem. However, it has recently been reported that auditory difficulties or decreases in body temperature caused by local or systemic administration of TRPA1 antagonists did not occur [29, 37]. It is still controversial whether inactivation of TRPA1 results in decreasing response to cold stimuli [7].

The present study showed that a TRPA1 antagonist suppressed transmission of physiological pain and neuropathic pain in the spinal dorsal horn. These findings indicate that TRPA1 antagonists can be used as universal analgesic drugs. On the other hand, the TRPA1 antagonist also decreased EPSCs evoked by air-puff stimuli in naïve rats. Therefore, it is possible that transmission of normal tactile stimuli or warning signals to the body may be inhibited as a side effect.

## Conclusion

In the present study, we found that a TRPA1 antagonist had an inhibitory effect on mechanical hypersensitivity and hyperalgesia as well as on non-noxious stimuli in the physiological state in the spinal cord dorsal horn. The results suggest that TRPA1 is consistently associated with excitability of SG neurons in the spinal cord dorsal horn and that it may have an important role in coordination of pain transmission.

## Methods

### Experimental animals

All experimental procedures were approved by the Animal Care and Use Committee of the Sapporo Medical University School of Medicine, Sapporo, Japan. We used a total of 220 male Sprague–Dawley rats aged 5 weeks and weighing 140–160 g at the beginning of the experiments. All efforts were made to minimize animal suffering and the number of animals used, and the experiments followed the ethical guidelines of the International Association for the Study of Pain [38].

### Surgical procedures for producing the radicular pain model

The animals were randomly divided into 3 experimental groups: the RC group, sham operation group, and naïve group. The surgical procedures have been described in detail previously [39]. In brief, after the rats were anesthetized with intraperitoneal sodium pentobarbital (50 mg/kg), they were placed in the prone position, and a midline dorsal incision was made in the lower back to expose the laminas between the L4 and S1 vertebrae. The paraspinal muscles were retracted to expose the right L5–L6 facet joint. A right L5 hemilaminectomy and an L5–L6 partial facetectomy were performed. In the RC group, the right L5 spinal root was carefully exposed and tightly ligated extradurally by using 8–0 nylon sutures proximal to DRG. The subcutaneous tissue and skin were then closed by using 4–0 nylon sutures. In the RC group, 78 rats (n = 15 for the behavioral studies, n = 63 for the electrophysiological studies, as described below) and in the sham operation group, 62 rats (n = 15 for the behavioral studies, n = 47 for the electrophysiological studies, as described below) underwent the surgical procedure as described above without nerve root constriction. In the naïve group, 80 rats (n = 15 for the behavioral studies, n = 65 for the electrophysiological studies, as described below) were used with any surgical procedures.

### Evaluation of mechanical hypersensitivity

To assess the mechanical withdrawal response, the rats were placed in a Plexiglas chamber (IITC Life Science Inc., Woodland Hills, CA, USA, measuring 18 × 25 × 18 cm above a wire mesh floor, which allowed full access to the hind paw, and were acclimatized to the environment for at least 20 minutes before the test. A 3.2-g von Frey filament (Semmes–Weinstein Monofilaments, North Coast Medical Inc., San Jose, CA, USA) was used to produce mechanical tactile stimuli, which were applied to the middle area between the foot pads on the plantar surface of the right (constriction side) and left (contralateral side) hind paws. Each hind paw was probed consecutively by 10 stimulations alternating between the right and left for each set. This was repeated thrice at intervals of at least 10 minutes [39, 40]. Mechanical sensitivity was evaluated as the response rate of withdrawal responses. Visible lifting of the stimulated hind limb was considered to be a withdrawal response. This procedure was performed 1 day before and 3, 5, 7, 10 and 14 days after surgery.

### Evaluation of physiological pain and mechanical hyperalgesia

Followed by the von Frey test, a pin-prick test was used to evaluate mechanical nociception and hyperalgesia [23]. A 26-gauge needle was used to apply noxious pain stimuli in the same manner as used for the von Frey filament. The needle was pressed up to 0.5 g to prevent tissue damage. The response rate of each rat were expressed at the injured side and noninjured side, respectively.

### Evaluation of thermal hyperalgesia

To assess the thermal withdrawal response, the rats were placed in a Plexiglas chamber on a glass platform and allowed to acclimatize for at least 20 minutes before the test. The thermal withdrawal response was measured as the latency of hind paw withdrawals elicited by a radiant heat source (Tail Flick Analgesia Meter, IITC Life Science Inc.), which was moved beneath the portion of the hind paw that was flush against the glass, and thermal stimulation was delivered to that site. Thermal hyperalgesia was evaluated as thermal withdrawal latency, which was defined as the time from the onset of radiant heat to the withdrawal of the tested foot. A cutoff time of 10 seconds was set to prevent tissue damage. Each hind paw was tested 5 times with at least 5-minute intervals, alternating between the right and left. The mean withdrawal latency was calculated from the last 4 measurements. Consequently, the thermal withdrawal latency of each rat was defined as the differential score, which was calculated by subtracting the withdrawal latency of the injured side from that of the noninjured side. Positive and negative scores indicated increased and decreased sensitivity of the ipsilateral hind paw, respectively. This test was performed after evaluating mechanical sensitivity and hyperalgesia.

### Estimation of spontaneous pain-related behaviors

To assess spontaneous pain, the rats were placed in a Plexiglas chamber above a wire mesh floor and allowed to acclimatize for 20 minutes before the test. Then, their behaviors were observed for 5 minutes. A numerical scale, as modified from a method described previously [4, 41], was used to evaluate the degree of spontaneous behavior related to spontaneous pain. The following scale was used in this study: 0 = the paw of the operated side was pressed normally on the floor; 1 = the paw rested lightly on the floor; 2 = only the internal edge of the paw was pressed on the floor; 3 = only the heel was pressed on the floor, and the hind paw was in an inverted position; 4 = the whole paw was elevated; 5 = the animal licked the lesioned paw. The largest scale number was adopted to estimate the spontaneous pain-related behavior of the tested rat. This test was performed in 14 days after surgery when mechanical hypersensitivity, hyperalgesia, and thermal hyperalgesia had fully developed. Blinding procedures were used in these behavioral experiments.

### Preparation for intrathechal drug administrations

For intrathecal drug injections, rats were anesthetized with inhalation of isoflurane (1.5%–2%), and a polyethylene catheter (PE-10; Clay Adams, Parsippany, NJ) filled with saline was slowly inserted through an opening in the cisterna magna into the spinal subarachnoid space [42–44] so that its tip lay at the level of the lumbar enlargement of the spinal cord. This procedure was performed after the behavioral evaluation in 14 days after surgery. The external part of the catheter was tunneled subcutaneously to exit on the top of the skull and plugged with welting at the end of the catheter. The position of the catheter was confirmed in the intrathecal space at the lumbar enlargement by exposing the lumbar spinal cord after sacrificing the animals by administering a pentobarbital overdose at the end of the study. The data from rats in which the catheter was in an inappropriate position were excluded from this study.

### Effects of the selective TRPA1 antagonist on pain-related behavior

In 15 days after surgery (within 24 hours after inserting the catheter), we performed the von Frey test described above for confirming that the catheter-implanted rats showed no evident motor disturbances such as paralysis. A selective antagonist for TRPA1, HC-030031 (Sigma, St Louis, MO, USA), was used to evaluate the involvement of TRPA1 in mechanical hyperalgesia. The rats were treated with HC-030031 (10 μg/site) or with vehicle [10% dimethylsulfoxide (DMSO) in saline] intrathecally. The mechanical hypersensitivity and hyperalgesia were evaluated using the von Frey test and pin-prick test described above at different time points after HC-030031 treatment (5, 15, and 30, 60, 120, 240 and 480 minutes), and spontaneous pain was estimated using the spontaneous pain scale described above at different time points after HC-030031 treatment (30 minutes and 2 hours). For the von Frey test and pin-prick test, the effects of HC-030031 was assessed as the response rate. Furthermore, the effect was compared with the intrathecal injection efficacy, which was calculated by dividing the withdrawal frequency after HC-030031 treatment by the baseline frequency.

### Electrophysiological procedures

We performed *in vivo* patch-clamp recording according to a previously described method [2, 4, 45–48]. The rats were used 11–14 days after surgery when mechanical hypersensitivity and thermal hyperalgesia had fully developed. The examiner was blinded to the 3 groups. The rats were supplied oxygen through a nose cone under anesthesia with intraperitoneal urethane (1.2–1.5 g/kg). Rectal temperature was maintained at 37–38°C using a heating pad. Laminectomy was performed from the T13– L2 levels, after which the rats were placed in a stereotaxic apparatus (Model ST-7, Narishige Group Co, Tokyo, Japan), and the spinal cord was carefully exposed. After removing the dura, the right L5 nerve root was observed microscopically as a swollen area with a darker color than the left L5 and right L4 nerve roots. The pia-arachnoid membranes of the right L5 dorsal root entry zone were cut to create a window large enough to insert the patch electrode. The surface of the spinal cord was irrigated with 95% O_2_ 5% CO_2_-equilibrated Krebs solution (10–15 ml/min) (in mM): 117 NaCl, 3.6 KCl, 2.5 CaCl_2_, 1.2 MgCl_2_, 1.2 NaH_2_PO_4_, 11 glucose, and 25 NaHCO_3_ at 37.0 ± 0.5°C.

The patch electrodes were pulled from thin-walled borosilicate glass capillaries (outer diameter of 1.5 mm, TW150F-4, World Precision Instruments, Sarasota, FL, USA) by using a puller (p-97, Sutter Instruments, Novato, CA, USA) and were filled with patch-pipette solutions composed of the following (in mM): 135 K-gluconate, 5 KCl, 0.5 CaCl_2_, 2 MgCl_2_, 5 EGTA, 5 ATP-Mg, 5 HEPES–KOH (pH 7.2) for the EPSC recordings and current-clamp recordings. The recording electrode (resistance range, 8–12 MΩ) was advanced at an angle of 30° into SG. After a gigaohm seal was formed with neurons at a depth of 50–150 μm from the surface of the spinal cord, the membrane patch was ruptured by negative pressure, and then the whole-cell patch-clamp recording was initiated. A patch-clamp amplifier (Axopatch 200B, Axon Instruments, Union City, CA, USA) was used to make recordings. In the voltage-clamp mode, the holding potentials (V_H_) were -70 mV, at which glycine-mediated and gamma-aminobutyric acid-mediated inhibitory postsynaptic currents were negligible [49]. Spontaneous excitatory postsynaptic potentials and action potentials were recorded in the current-clamp mode. Spontaneous and evoked EPSCs were recorded as described later. Whole-cell patch-clamp recordings were made from SG neurons at the L5 segment of the spinal cord in the RC group, sham group, and naïve group. Data from neurons with resting membrane potentials more positive than -50 mV were excluded from this study.

After the electrophysiological experiment, some neurons that have been injected with biocytin during the recording were confirmed to be located in SG as described previously [2, 4, 45, 47]. An A/D converter (Digidata 1322A, Axon Instruments) was used to digitize the data, which were analyzed by using the Mini Analysis Program version 6.0.7 (Synaptosoft, Fort Lee, NJ, USA).

In the voltage-clamp mode, all SG neurons produced large amplitude (>50 pA) barrages of EPSCs to non-noxious or noxious stimuli, such as a paintbrush or toothed forceps to the skin of the lumbar and pelvic regions or the hind paw [2, 4, 45, 46]. A paintbrush or toothed forceps applied to the skin of the abdominal wall, lumbar and pelvic regions, hind paw, and tail was used to carefully determine the receptive field of a neuron after repeated stimuli. If evoked EPSCs were not observed during stimulation, that neuron was defined as one with no receptive field.

After determining the receptive field, the responses to non-noxious stimuli were assessed using a puff of air (20 psi) through a pipette (outer diameter of 200 μm) applied repetitively to the center of the receptive field (duration of injection, 5 seconds) using a pico-injector (PLI-100, Harvard Apparatus, Holliston, MA, USA), according to a previously described method [4, 47]. The air-puff stimuli did not cause pain to the examiners. The noxious pinch stimuli were applied with toothed forceps that were firmly fixed on a rod. A weight (60 g) was placed on the forceps for 5 seconds, as referred to in previous reports [4, 45, 47]. SG neurons exhibited several response profiles during air-puff or pinch stimuli in the current-clamp mode [50, 51], which have been described in detail previously [4, 47]. If the recorded neurons responded maximally to non-noxious stimuli enough to reach the action potential threshold, they were excluded in the present study because they may have been located in the lamina or deeper [4]. The air-puff and pinch stimuli were repeated thrice to evaluate the evoked activities of neurons. Then mean frequencies and amplitudes of the evoked EPSCs for each SG neuron during the stimulation were calculated by the values of the 3 records.

### Drug application

Drug was dissolved in Krebs solution and applied by perfusion via a 3-way stopcock without any change in the perfusion rate or the temperature [48]. The time necessary for the solution to flow from the stopcock to the surface of the spinal cord was approximately 30 seconds. The drug used in this study was HC-030031 (Waco, Osaka, Japan). HC-030031 was first dissolved in DMSO at 1000 times the concentrations to be used and then was diluted to the final concentration in Krebs solution immediately before use.

### Statistical analysis

All numerical data are expressed as means ± SEMs. One-way factorial measures of analysis of variance followed by the Tukey–Kramer test were used to analyze the behavioral test data and electrophysiological data of the 3 groups. Student’s paired *t* test, the Mann–Whitney *U* test, and the Wilcoxon signed-ranks test were used for analysis of changes in parameters between the 2 groups. The level of statistical significance was set to P < 0.05.

## Abbreviations

DRG: Dorsal root ganglion
DMSO: Dimethylsulfoxide
EPSC: Excitatory postsynaptic current
RC: Root constriction
SG: Substantia gelatinosa
SNL: Spinal nerve ligation
TRP: Transient receptor potential
TRPV1: Transient receptor potential vanilloid 1
TRPA1: Transient receptor potential ankyrin 1.
